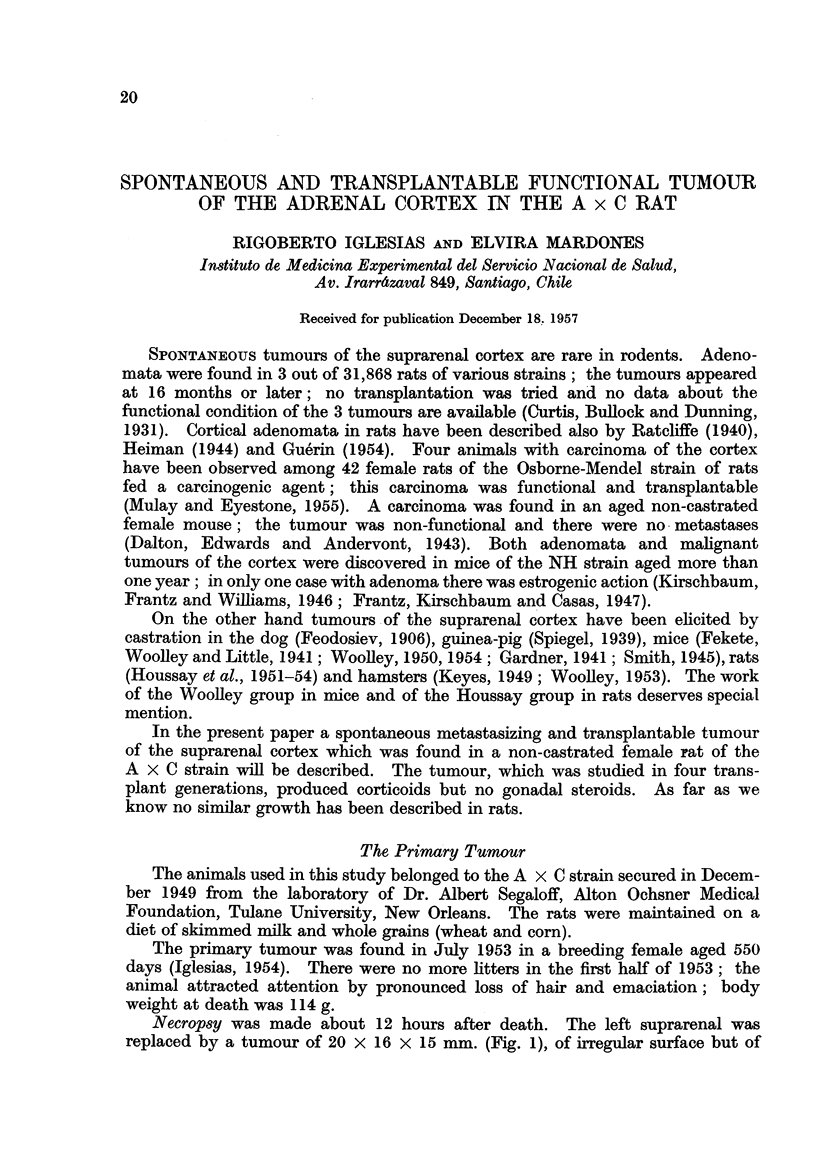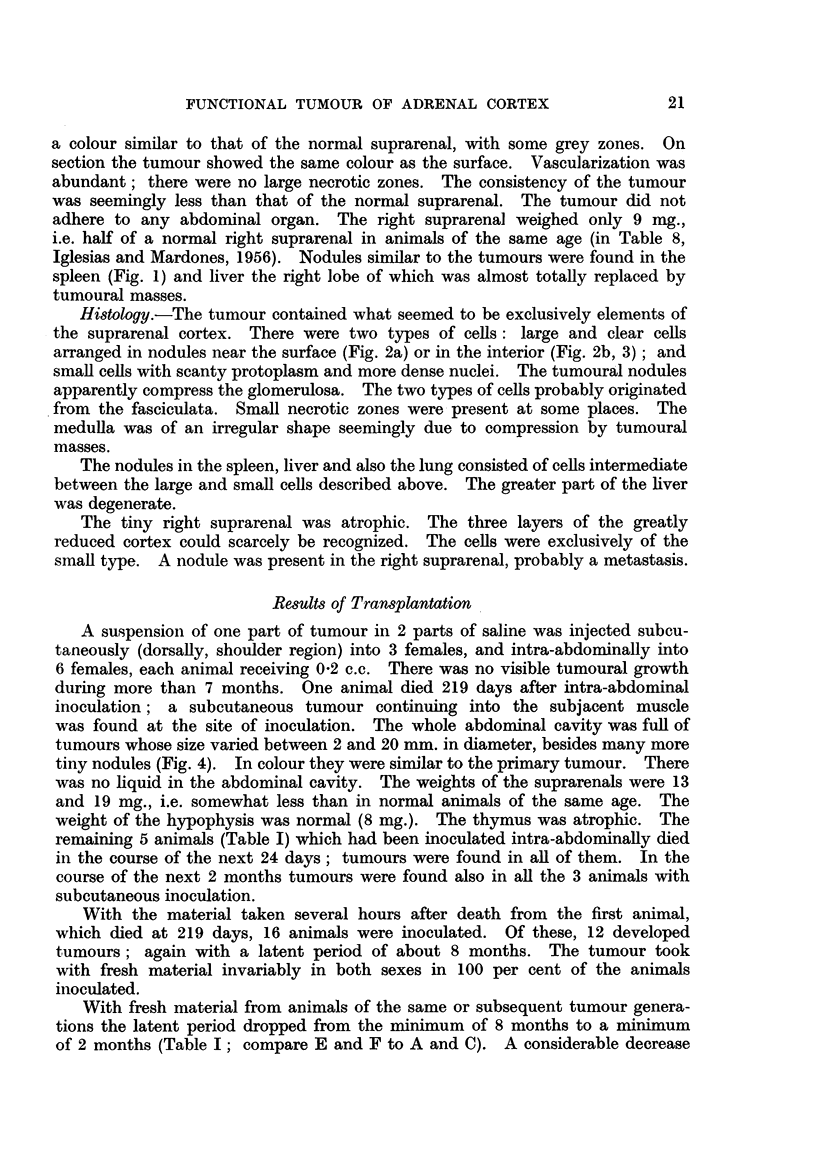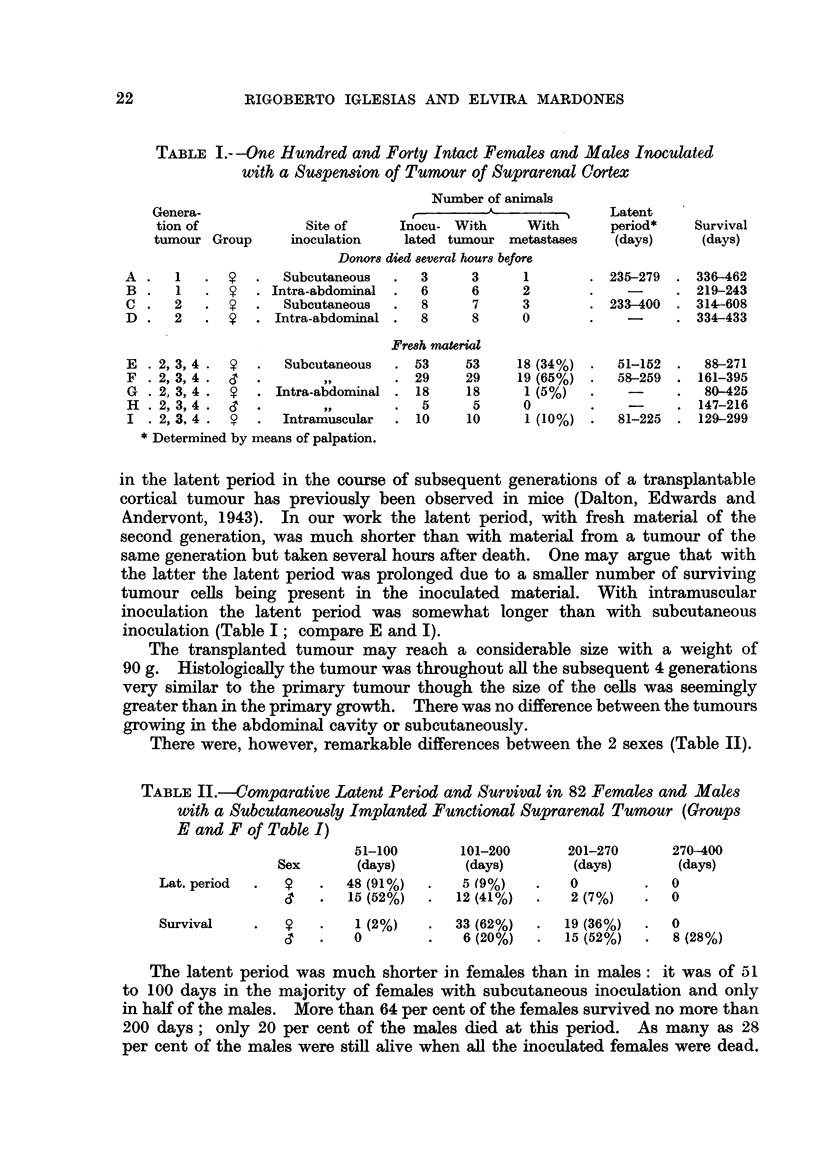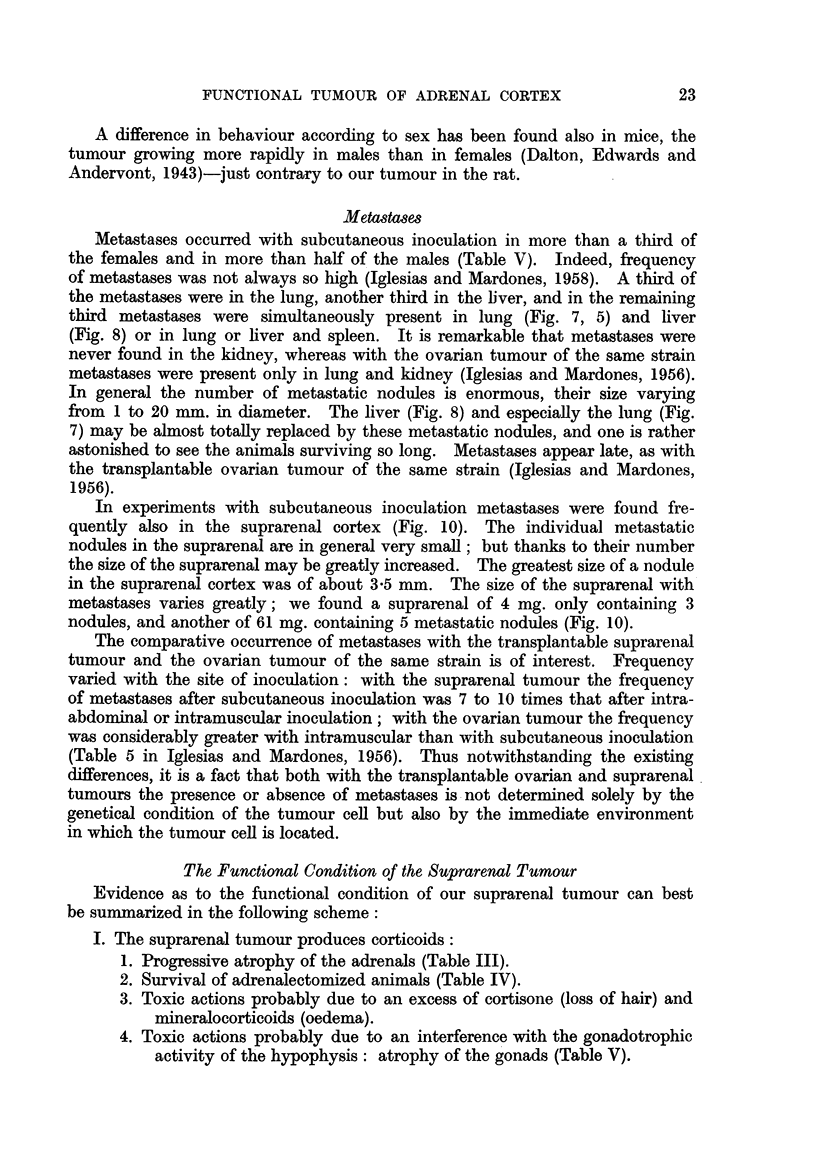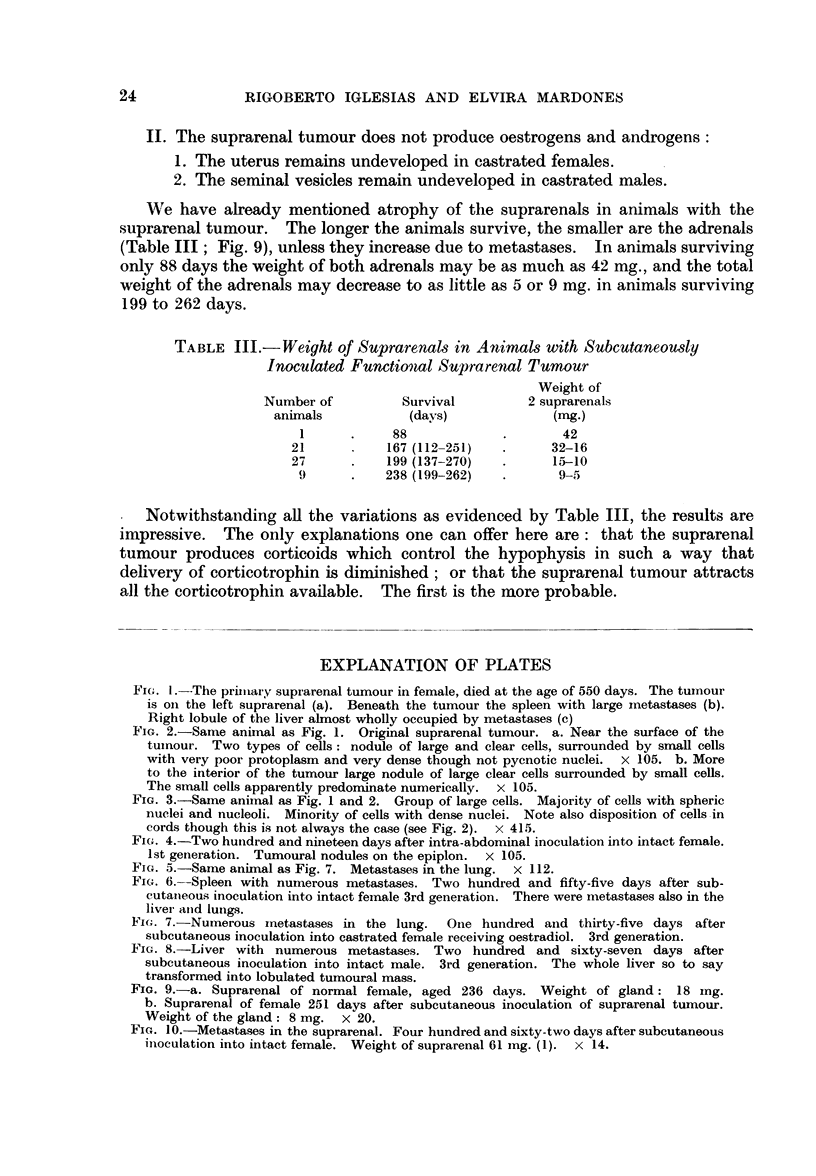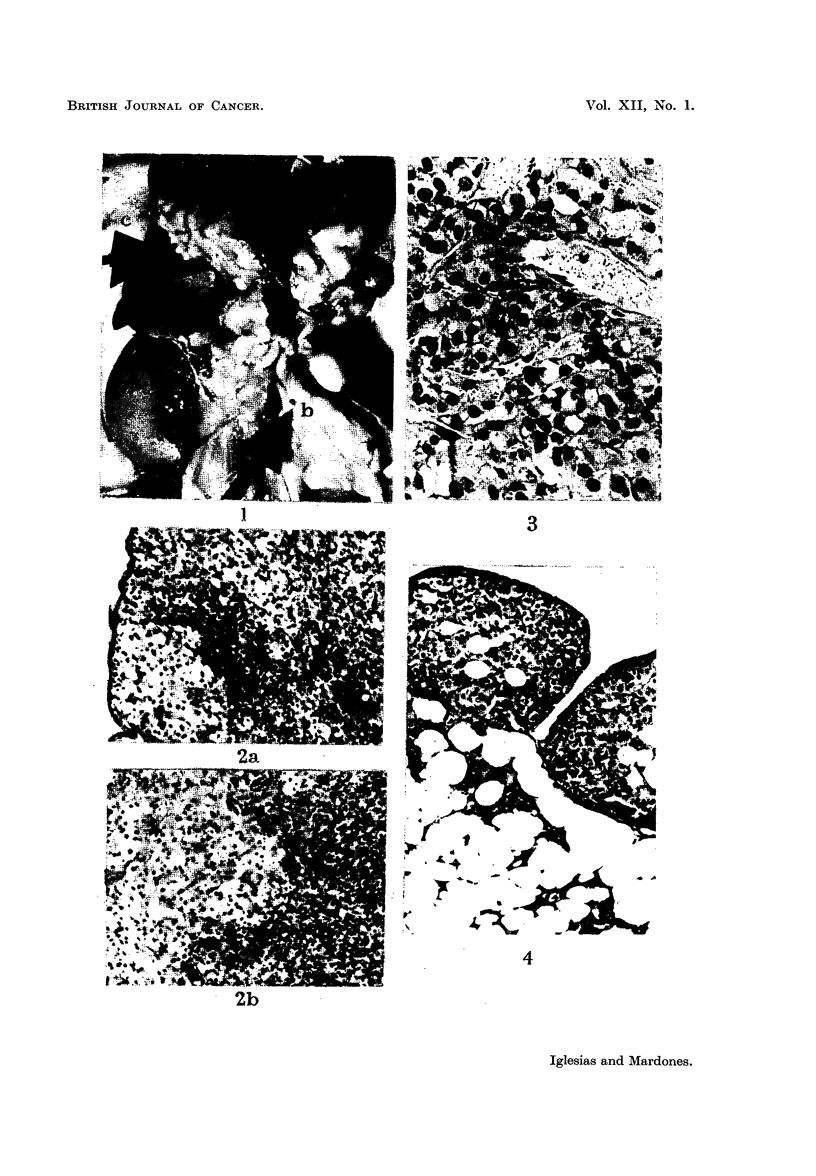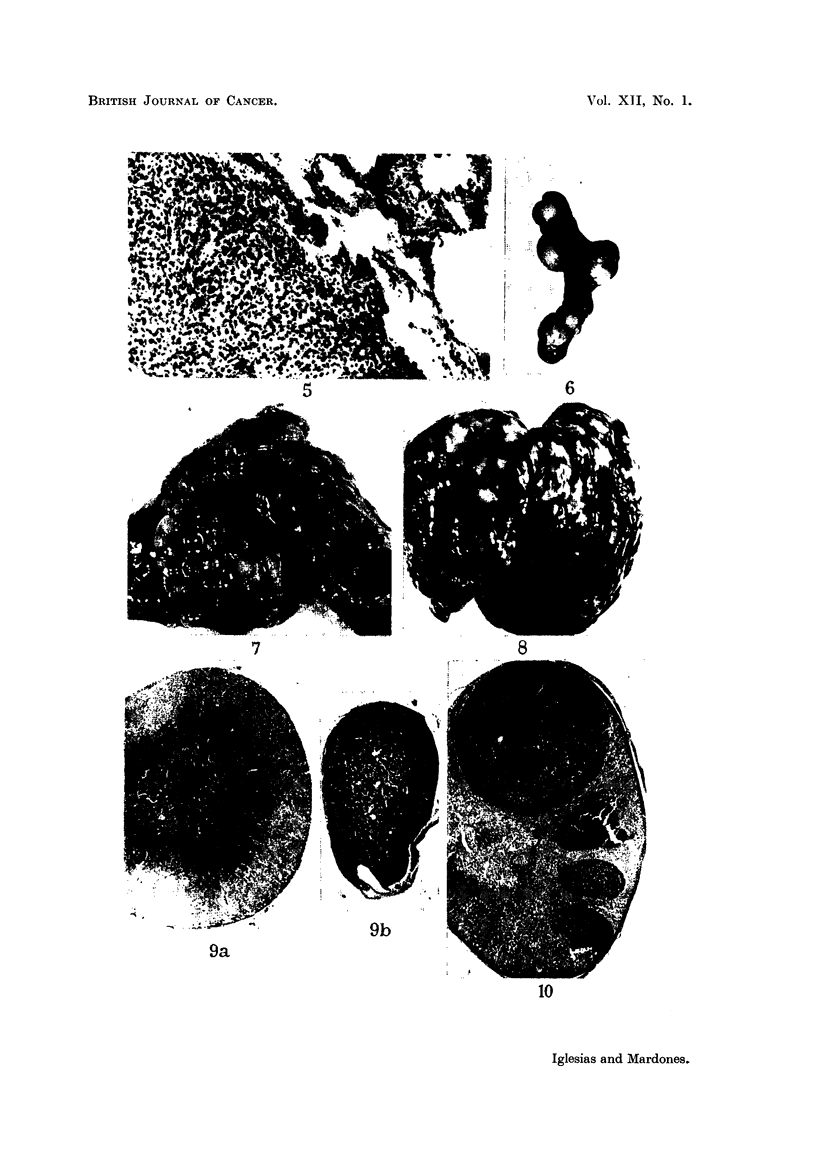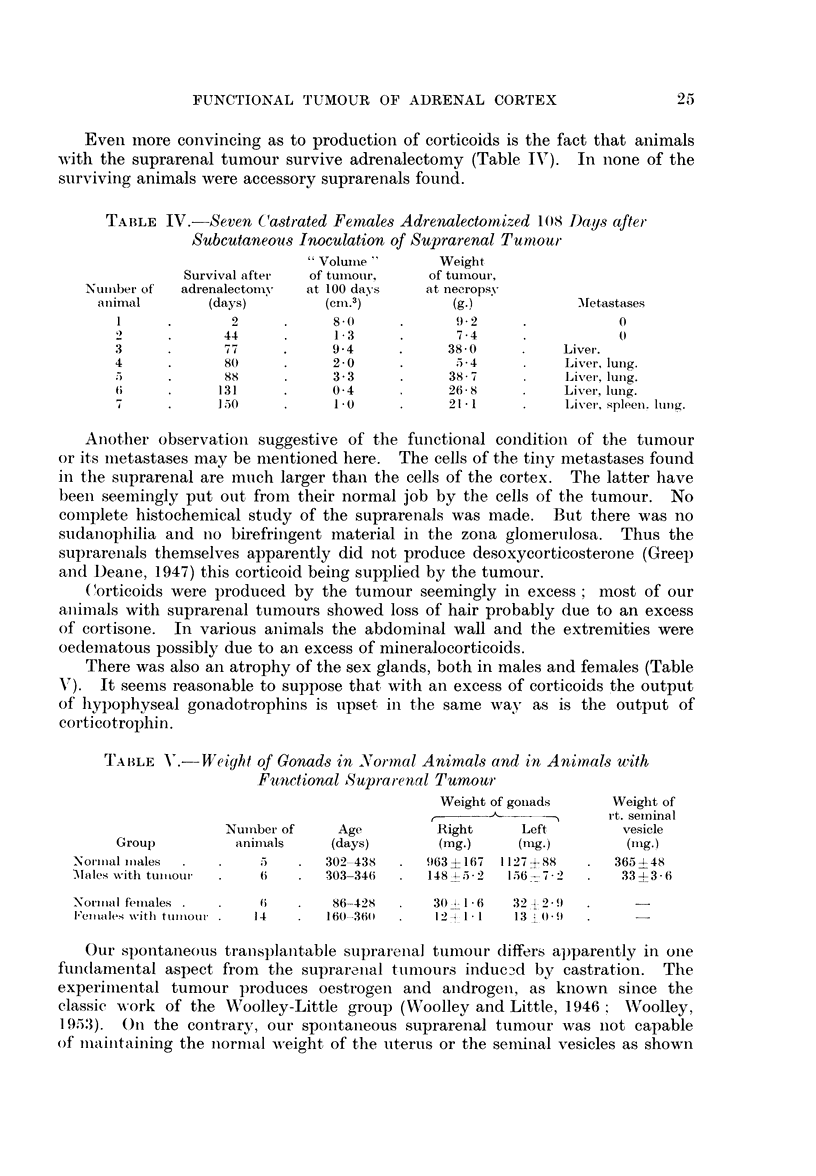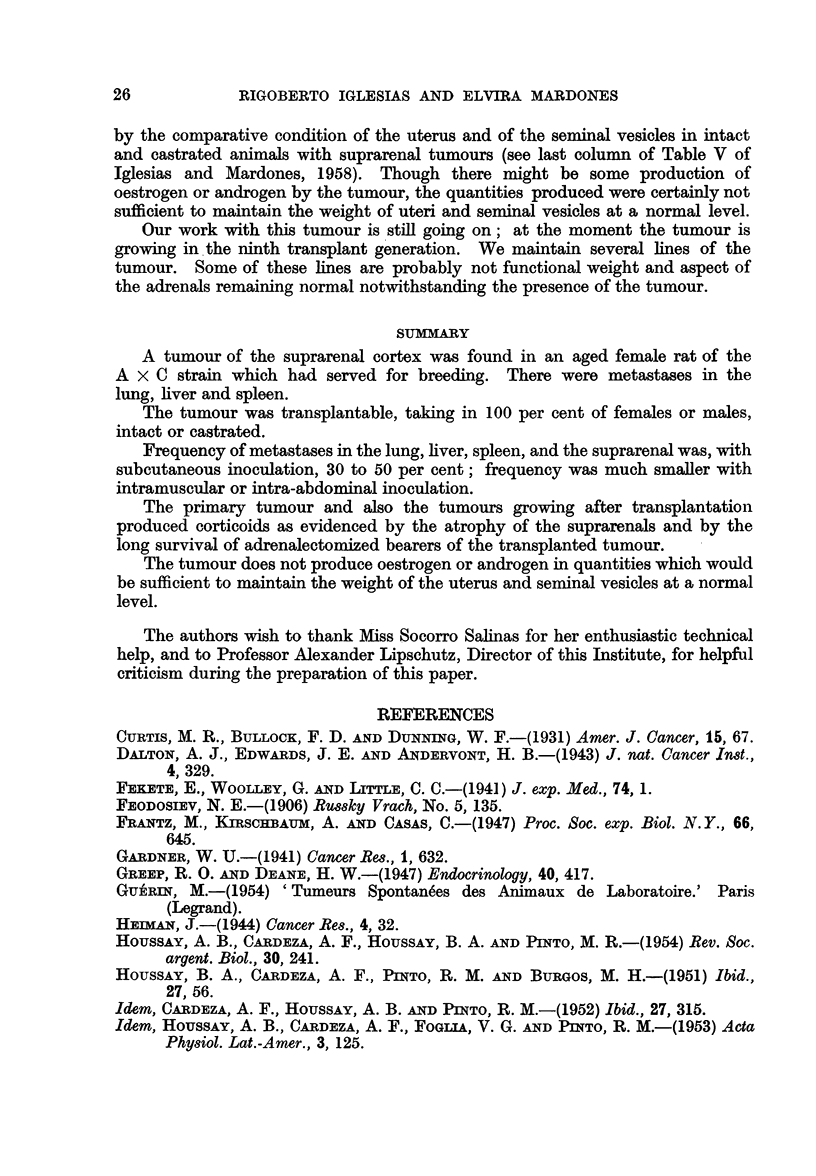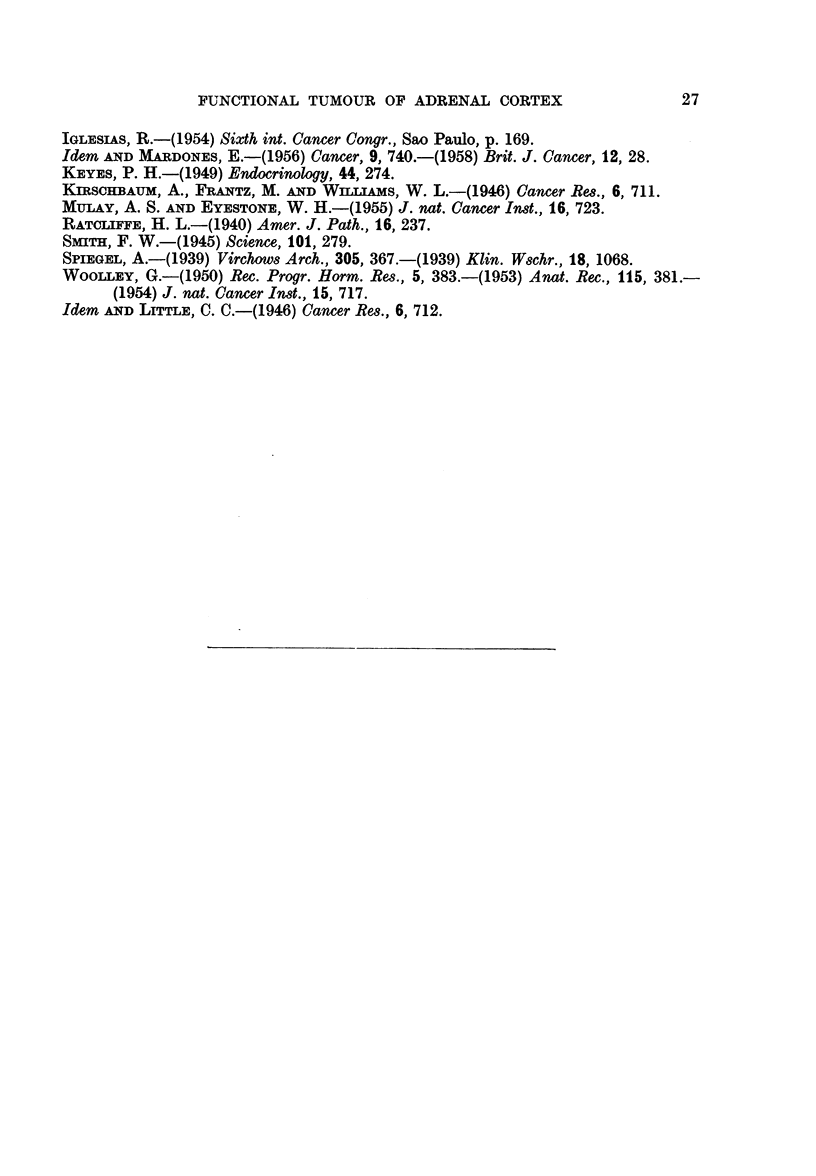# Spontaneous and Transplantable Functional Tumour of the Adrenal Cortex in the A × C Rat

**DOI:** 10.1038/bjc.1958.4

**Published:** 1958-03

**Authors:** Rigoberto Iglesias, Elvira Mardones

## Abstract

**Images:**


### SPONTANEOUS AND TRANSPLANTABLE FUNCTIONAL TUMOUR OF THE ADRENAL CORTEX IN THE A × C RAT

RIGOBERTO IGLESIAS AND ELVIRA MARDONES
*Instituto de Medicina Experimental del Servicio Nacional de Salud, Av. Irarrázaval 849, Santiago, Chile*

Received for publication December 18, 1957

SPONTANEOUS tumours of the suprarenal cortex are rare in rodents. Adenomata were found in 3 out of 31,868 rats of various strains ; the tumours appeared at 16 months or later ; no transplantation was tried and no data about the functional condition of the 3 tumours are available (Curtis, Bullock and Dunning, 1931). Cortical adenomata in rats have been described also by Ratcliffe (1940), Heiman (1944) and Guérin (1954). Four animals with carcinoma of the cortex have been observed among 42 female rats of the Osborne-Mendel strain of rats fed a carcinogenic agent ; this carcinoma was functional and transplantable (Mulay and Eyestone, 1955). A carcinoma was found in an aged non-castrated female mouse ; the tumour was non-functional and there were no metastases (Dalton, Edwards and Andervont, 1943). Both adenomata and malignant tumours of the cortex were discovered in mice of the NH strain aged more than one year ; in only one case with adenoma there was estrogenic action (Kirschbaum, Frantz and Williams, 1946 ; Frantz, Kirschbaum and Casas, 1947).

On the other hand tumours of the suprarenal cortex have been elicited by castration in the dog (Feodosiev, 1906), guinea-pig (Spiegel, 1939), mice (Fekete, Woolley and Little, 1941 ; Woolley, 1950, 1954 ; Gardner, 1941 ; Smith, 1945), rats (Houssay *et al.*, 1951–54) and hamsters (Keyes, 1949 ; Woolley, 1953). The work of the Woolley group in mice and of the Houssay group in rats deserves special mention.

In the present paper a spontaneous metastasizing and transplantable tumour of the suprarenal cortex which was found in a non-castrated female rat of the A × C strain will be described. The tumour, which was studied in four transplant generations, produced corticoids but no gonadal steroids. As far as we know no similar growth has been described in rats.

### *The Primary Tumour*

The animals used in this study belonged to the A × C strain secured in December 1949 from the laboratory of Dr. Albert Segaloff, Alton Ochsner Medical Foundation, Tulane University, New Orleans. The rats were maintained on a diet of skimmed milk and whole grains (wheat and corn).

The primary tumour was found in July 1953 in a breeding female aged 550 days (Iglesias, 1954). There were no more litters in the first half of 1953 ; the animal attracted attention by pronounced loss of hair and emaciation ; body weight at death was 114 g.

*Necropsy* was made about 12 hours after death. The left suprarenal was replaced by a tumour of 20 × 16 × 15 mm. (Fig. 1), of irregular surface but of a colour similar to that of the normal suprarenal, with some grey zones. On section the tumour showed the same colour as the surface. Vascularization was abundant ; there were no large necrotic zones. The consistency of the tumour was seemingly less than that of the normal suprarenal. The tumour did not adhere to any abdominal organ. The right suprarenal weighed only 9 mg., i.e. half of a normal right suprarenal in animals of the same age (in Table 8, Iglesias and Mardones, 1956). Nodules similar to the tumours were found in the spleen (Fig. 1) and liver the right lobe of which was almost totally replaced by tumoural masses.

*Histology.*—The tumour contained what seemed to be exclusively elements of the suprarenal cortex. There were two types of cells : large and clear cells arranged in nodules near the surface (Fig. 2a) or in the interior (Fig. 2b, 3) ; and small cells with scanty protoplasm and more dense nuclei. The tumoural nodules apparently compress the glomerulosa. The two types of cells probably originated from the fasciculata. Small necrotic zones were present at some places. The medulla was of an irregular shape seemingly due to compression by tumoural masses.

The nodules in the spleen, liver and also the lung consisted of cells intermediate between the large and small cells described above. The greater part of the liver was degenerate.

The tiny right suprarenal was atrophic. The three layers of the greatly reduced cortex could scarcely be recognized. The cells were exclusively of the small type. A nodule was present in the right suprarenal, probably a metastasis.

### *Results of Transplantation*

A suspension of one part of tumour in 2 parts of saline was injected subcutaneously (dorsally, shoulder region) into 3 females, and intra-abdominally into 6 females, each animal receiving 0·2 c.c. There was no visible tumoural growth during more than 7 months. One animal died 219 days after intra-abdominal inoculation ; a subcutaneous tumour continuing into the subjacent muscle was found at the site of inoculation. The whole abdominal cavity was full of tumours whose size varied between 2 and 20 mm. in diameter, besides many more tiny nodules (Fig. 4). In colour they were similar to the primary tumour. There was no liquid in the abdominal cavity. The weights of the suprarenals were 13 and 19 mg., i.e. somewhat less than in normal animals of the same age. The weight of the hypophysis was normal (8 mg.). The thymus was atrophic. The remaining 5 animals (Table I) which had been inoculated intra-abdominally died in the course of the next 24 days ; tumours were found in all of them. In the course of the next 2 months tumours were found also in all the 3 animals with subcutaneous inoculation.

With the material taken several hours after death from the first animal, which died at 219 days, 16 animals were inoculated. Of these, 12 developed tumours ; again with a latent period of about 8 months. The tumour took with fresh material invariably in both sexes in 100 per cent of the animals inoculated.

With fresh material from animals of the same or subsequent tumour generations the latent period dropped from the minimum of 8 months to a minimum of 2 months (Table I ; compare E and F to A and C). A considerable decrease

TABLE I.—*One Hundred and Forty Intact Females and Males Inoculated with a Suspension of Tumour of Suprarenal Cortex*

| | Generation of tumour | Group | Site of inoculation | Number of animals: Inoculated | With tumour | With metastases | Latent period* (days) | Survival (days) |
|---|---|---|---|---|---|---|---|---|
| | | | *Donors died several hours before* | | | | | |
| A | 1 | ♀ | Subcutaneous | 3 | 3 | 1 | 235–279 | 336–462 |
| B | 1 | ♀ | Intra-abdominal | 6 | 6 | 2 | — | 219–243 |
| C | 2 | ♀ | Subcutaneous | 8 | 7 | 3 | 233–400 | 314–608 |
| D | 2 | ♀ | Intra-abdominal | 8 | 8 | 0 | — | 334–433 |
| | | | *Fresh material* | | | | | |
| E | 2, 3, 4 | ♀ | Subcutaneous | 53 | 53 | 18 (34%) | 51–152 | 88–271 |
| F | 2, 3, 4 | ♂ | ,, | 29 | 29 | 19 (65%) | 58–259 | 161–395 |
| G | 2, 3, 4 | ♀ | Intra-abdominal | 18 | 18 | 1 (5%) | — | 80–425 |
| H | 2, 3, 4 | ♂ | ,, | 5 | 5 | 0 | — | 147–216 |
| I | 2, 3, 4 | ♀ | Intramuscular | 10 | 10 | 1 (10%) | 81–225 | 129–299 |

* Determined by means of palpation.

in the latent period in the course of subsequent generations of a transplantable cortical tumour has previously been observed in mice (Dalton, Edwards and Andervont, 1943). In our work the latent period, with fresh material of the second generation, was much shorter than with material from a tumour of the same generation but taken several hours after death. One may argue that with the latter the latent period was prolonged due to a smaller number of surviving tumour cells being present in the inoculated material. With intramuscular inoculation the latent period was somewhat longer than with subcutaneous inoculation (Table I; compare E and I).

The transplanted tumour may reach a considerable size with a weight of 90 g. Histologically the tumour was throughout all the subsequent 4 generations very similar to the primary tumour though the size of the cells was seemingly greater than in the primary growth. There was no difference between the tumours growing in the abdominal cavity or subcutaneously.

There were, however, remarkable differences between the 2 sexes (Table II).

TABLE II.—*Comparative Latent Period and Survival in* 82 *Females and Males with a Subcutaneously Implanted Functional Suprarenal Tumour* (*Groups E and F of Table I*)

| | Sex | 51–100 (days) | 101–200 (days) | 201–270 (days) | 270–400 (days) |
|---|---|---|---|---|---|
| Lat. period | ♀ | 48 (91%) | 5 (9%) | 0 | 0 |
| | ♂ | 15 (52%) | 12 (41%) | 2 (7%) | 0 |
| Survival | ♀ | 1 (2%) | 33 (62%) | 19 (36%) | 0 |
| | ♂ | 0 | 6 (20%) | 15 (52%) | 8 (28%) |

The latent period was much shorter in females than in males: it was of 51 to 100 days in the majority of females with subcutaneous inoculation and only in half of the males. More than 64 per cent of the females survived no more than 200 days; only 20 per cent of the males died at this period. As many as 28 per cent of the males were still alive when all the inoculated females were dead.

A difference in behaviour according to sex has been found also in mice, the tumour growing more rapidly in males than in females (Dalton, Edwards and Andervont, 1943)—just contrary to our tumour in the rat.

### *Metastases*

Metastases occurred with subcutaneous inoculation in more than a third of the females and in more than half of the males (Table V). Indeed, frequency of metastases was not always so high (Iglesias and Mardones, 1958). A third of the metastases were in the lung, another third in the liver, and in the remaining third metastases were simultaneously present in lung (Fig. 7, 5) and liver (Fig. 8) or in lung or liver and spleen. It is remarkable that metastases were never found in the kidney, whereas with the ovarian tumour of the same strain metastases were present only in lung and kidney (Iglesias and Mardones, 1956). In general the number of metastatic nodules is enormous, their size varying from 1 to 20 mm. in diameter. The liver (Fig. 8) and especially the lung (Fig. 7) may be almost totally replaced by these metastatic nodules, and one is rather astonished to see the animals surviving so long. Metastases appear late, as with the transplantable ovarian tumour of the same strain (Iglesias and Mardones, 1956).

In experiments with subcutaneous inoculation metastases were found frequently also in the suprarenal cortex (Fig. 10). The individual metastatic nodules in the suprarenal are in general very small; but thanks to their number the size of the suprarenal may be greatly increased. The greatest size of a nodule in the suprarenal cortex was of about 3·5 mm. The size of the suprarenal with metastases varies greatly; we found a suprarenal of 4 mg. only containing 3 nodules, and another of 61 mg. containing 5 metastatic nodules (Fig. 10).

The comparative occurrence of metastases with the transplantable suprarenal tumour and the ovarian tumour of the same strain is of interest. Frequency varied with the site of inoculation: with the suprarenal tumour the frequency of metastases after subcutaneous inoculation was 7 to 10 times that after intra-abdominal or intramuscular inoculation; with the ovarian tumour the frequency was considerably greater with intramuscular than with subcutaneous inoculation (Table 5 in Iglesias and Mardones, 1956). Thus notwithstanding the existing differences, it is a fact that both with the transplantable ovarian and suprarenal tumours the presence or absence of metastases is not determined solely by the genetical condition of the tumour cell but also by the immediate environment in which the tumour cell is located.

### *The Functional Condition of the Suprarenal Tumour*

Evidence as to the functional condition of our suprarenal tumour can best be summarized in the following scheme:

I. The suprarenal tumour produces corticoids:
   1. Progressive atrophy of the adrenals (Table III).
   2. Survival of adrenalectomized animals (Table IV).
   3. Toxic actions probably due to an excess of cortisone (loss of hair) and mineralocorticoids (oedema).
   4. Toxic actions probably due to an interference with the gonadotrophic activity of the hypophysis: atrophy of the gonads (Table V).

II. The suprarenal tumour does not produce oestrogens and androgens :

1. The uterus remains undeveloped in castrated females.
2. The seminal vesicles remain undeveloped in castrated males.

We have already mentioned atrophy of the suprarenals in animals with the suprarenal tumour. The longer the animals survive, the smaller are the adrenals (Table III ; Fig. 9), unless they increase due to metastases. In animals surviving only 88 days the weight of both adrenals may be as much as 42 mg., and the total weight of the adrenals may decrease to as little as 5 or 9 mg. in animals surviving 199 to 262 days.

TABLE III.—*Weight of Suprarenals in Animals with Subcutaneously Inoculated Functional Suprarenal Tumour*

| Number of animals | Survival (days) | Weight of 2 suprarenals (mg.) |
|---|---|---|
| 1 | 88 | 42 |
| 21 | 167 (112–251) | 32–16 |
| 27 | 199 (137–270) | 15–10 |
| 9 | 238 (199–262) | 9–5 |

Notwithstanding all the variations as evidenced by Table III, the results are impressive. The only explanations one can offer here are : that the suprarenal tumour produces corticoids which control the hypophysis in such a way that delivery of corticotrophin is diminished ; or that the suprarenal tumour attracts all the corticotrophin available. The first is the more probable.

### EXPLANATION OF PLATES

FIG. 1.—The primary suprarenal tumour in female, died at the age of 550 days. The tumour is on the left suprarenal (a). Beneath the tumour the spleen with large metastases (b). Right lobule of the liver almost wholly occupied by metastases (c)

FIG. 2.—Same animal as Fig. 1. Original suprarenal tumour. a. Near the surface of the tumour. Two types of cells : nodule of large and clear cells, surrounded by small cells with very poor protoplasm and very dense though not pycnotic nuclei. × 105. b. More to the interior of the tumour large nodule of large clear cells surrounded by small cells. The small cells apparently predominate numerically. × 105.

FIG. 3.—Same animal as Fig. 1 and 2. Group of large cells. Majority of cells with spheric nuclei and nucleoli. Minority of cells with dense nuclei. Note also disposition of cells in cords though this is not always the case (see Fig. 2). × 415.

FIG. 4.—Two hundred and nineteen days after intra-abdominal inoculation into intact female. 1st generation. Tumoural nodules on the epiplon. × 105.

FIG. 5.—Same animal as Fig. 7. Metastases in the lung. × 112.

FIG. 6.—Spleen with numerous metastases. Two hundred and fifty-five days after subcutaneous inoculation into intact female 3rd generation. There were metastases also in the liver and lungs.

FIG. 7.—Numerous metastases in the lung. One hundred and thirty-five days after subcutaneous inoculation into castrated female receiving oestradiol. 3rd generation.

FIG. 8.—Liver with numerous metastases. Two hundred and sixty-seven days after subcutaneous inoculation into intact male. 3rd generation. The whole liver so to say transformed into lobulated tumoural mass.

FIG. 9.—a. Suprarenal of normal female, aged 236 days. Weight of gland : 18 mg. b. Suprarenal of female 251 days after subcutaneous inoculation of suprarenal tumour. Weight of the gland : 8 mg. × 20.

FIG. 10.—Metastases in the suprarenal. Four hundred and sixty-two days after subcutaneous inoculation into intact female. Weight of suprarenal 61 mg. (1). × 14.

1

3

2a

2b

4

5

6

7

8

9a

9b

10

Even more convincing as to production of corticoids is the fact that animals with the suprarenal tumour survive adrenalectomy (Table IV). In none of the surviving animals were accessory suprarenals found.

TABLE IV.—*Seven Castrated Females Adrenalectomized* 108 *Days after Subcutaneous Inoculation of Suprarenal Tumour*

| Number of animal | Survival after adrenalectomy (days) | "Volume" of tumour, at 100 days (cm.$^3$) | Weight of tumour, at necropsy (g.) | Metastases |
|---|---|---|---|---|
| 1 | 2 | 8·0 | 9·2 | 0 |
| 2 | 44 | 1·3 | 7·4 | 0 |
| 3 | 77 | 9·4 | 38·0 | Liver. |
| 4 | 80 | 2·0 | 5·4 | Liver, lung. |
| 5 | 88 | 3·3 | 38·7 | Liver, lung. |
| 6 | 131 | 0·4 | 26·8 | Liver, lung. |
| 7 | 150 | 1·0 | 21·1 | Liver, spleen, lung. |

Another observation suggestive of the functional condition of the tumour or its metastases may be mentioned here. The cells of the tiny metastases found in the suprarenal are much larger than the cells of the cortex. The latter have been seemingly put out from their normal job by the cells of the tumour. No complete histochemical study of the suprarenals was made. But there was no sudanophilia and no birefringent material in the zona glomerulosa. Thus the suprarenals themselves apparently did not produce desoxycorticosterone (Greep and Deane, 1947) this corticoid being supplied by the tumour.

Corticoids were produced by the tumour seemingly in excess ; most of our animals with suprarenal tumours showed loss of hair probably due to an excess of cortisone. In various animals the abdominal wall and the extremities were oedematous possibly due to an excess of mineralocorticoids.

There was also an atrophy of the sex glands, both in males and females (Table V). It seems reasonable to suppose that with an excess of corticoids the output of hypophyseal gonadotrophins is upset in the same way as is the output of corticotrophin.

TABLE V.—*Weight of Gonads in Normal Animals and in Animals with Functional Suprarenal Tumour*

| Group | Number of animals | Age (days) | Weight of gonads: Right (mg.) | Weight of gonads: Left (mg.) | Weight of rt. seminal vesicle (mg.) |
|---|---|---|---|---|---|
| Normal males | 5 | 302–438 | 963 ± 167 | 1127 ± 88 | 365 ± 48 |
| Males with tumour | 6 | 303–346 | 148 ± 5·2 | 156 ± 7·2 | 33 ± 3·6 |
| Normal females | 6 | 86–428 | 30 ± 1·6 | 32 ± 2·9 | — |
| Females with tumour | 14 | 160–360 | 12 ± 1·1 | 13 ± 0·9 | — |

Our spontaneous transplantable suprarenal tumour differs apparently in one fundamental aspect from the suprarenal tumours induced by castration. The experimental tumour produces oestrogen and androgen, as known since the classic work of the Woolley-Little group (Woolley and Little, 1946 ; Woolley, 1953). On the contrary, our spontaneous suprarenal tumour was not capable of maintaining the normal weight of the uterus or the seminal vesicles as shown by the comparative condition of the uterus and of the seminal vesicles in intact and castrated animals with suprarenal tumours (see last column of Table V of Iglesias and Mardones, 1958). Though there might be some production of oestrogen or androgen by the tumour, the quantities produced were certainly not sufficient to maintain the weight of uteri and seminal vesicles at a normal level.

Our work with this tumour is still going on; at the moment the tumour is growing in the ninth transplant generation. We maintain several lines of the tumour. Some of these lines are probably not functional weight and aspect of the adrenals remaining normal notwithstanding the presence of the tumour.

### SUMMARY

A tumour of the suprarenal cortex was found in an aged female rat of the A × C strain which had served for breeding. There were metastases in the lung, liver and spleen.

The tumour was transplantable, taking in 100 per cent of females or males, intact or castrated.

Frequency of metastases in the lung, liver, spleen, and the suprarenal was, with subcutaneous inoculation, 30 to 50 per cent; frequency was much smaller with intramuscular or intra-abdominal inoculation.

The primary tumour and also the tumours growing after transplantation produced corticoids as evidenced by the atrophy of the suprarenals and by the long survival of adrenalectomized bearers of the transplanted tumour.

The tumour does not produce oestrogen or androgen in quantities which would be sufficient to maintain the weight of the uterus and seminal vesicles at a normal level.

The authors wish to thank Miss Socorro Salinas for her enthusiastic technical help, and to Professor Alexander Lipschutz, Director of this Institute, for helpful criticism during the preparation of this paper.

### REFERENCES

CURTIS, M. R., BULLOCK, F. D. AND DUNNING, W. F.—(1931) *Amer. J. Cancer*, **15**, 67.
DALTON, A. J., EDWARDS, J. E. AND ANDERVONT, H. B.—(1943) *J. nat. Cancer Inst.*, **4**, 329.
FEKETE, E., WOOLLEY, G. AND LITTLE, C. C.—(1941) *J. exp. Med.*, **74**, 1.
FEODOSIEV, N. E.—(1906) *Russky Vrach*, No. 5, 135.
FRANTZ, M., KIRSCHBAUM, A. AND CASAS, C.—(1947) *Proc. Soc. exp. Biol. N.Y.*, **66**, 645.
GARDNER, W. U.—(1941) *Cancer Res.*, **1**, 632.
GREEP, R. O. AND DEANE, H. W.—(1947) *Endocrinology*, **40**, 417.
GUÉRIN, M.—(1954) 'Tumeurs Spontanées des Animaux de Laboratoire.' Paris (Legrand).
HEIMAN, J.—(1944) *Cancer Res.*, **4**, 32.
HOUSSAY, A. B., CARDEZA, A. F., HOUSSAY, B. A. AND PINTO, M. R.—(1954) *Rev. Soc. argent. Biol.*, **30**, 241.
HOUSSAY, B. A., CARDEZA, A. F., PINTO, R. M. AND BURGOS, M. H.—(1951) *Ibid.*, **27**, 56.
*Idem*, CARDEZA, A. F., HOUSSAY, A. B. AND PINTO, R. M.—(1952) *Ibid.*, **27**, 315.
*Idem*, HOUSSAY, A. B., CARDEZA, A. F., FOGLIA, V. G. AND PINTO, R. M.—(1953) *Acta Physiol. Lat.-Amer.*, **3**, 125.

IGLESIAS, R.—(1954) *Sixth int. Cancer Congr.*, Sao Paulo, p. 169.
*Idem* AND MARDONES, E.—(1956) *Cancer*, **9**, 740.—(1958) *Brit. J. Cancer*, **12**, 28.
KEYES, P. H.—(1949) *Endocrinology*, **44**, 274.
KIRSCHBAUM, A., FRANTZ, M. AND WILLIAMS, W. L.—(1946) *Cancer Res.*, **6**, 711.
MULAY, A. S. AND EYESTONE, W. H.—(1955) *J. nat. Cancer Inst.*, **16**, 723.
RATCLIFFE, H. L.—(1940) *Amer. J. Path.*, **16**, 237.
SMITH, F. W.—(1945) *Science*, **101**, 279.
SPIEGEL, A.—(1939) *Virchows Arch.*, **305**, 367.—(1939) *Klin. Wschr.*, **18**, 1068.
WOOLLEY, G.—(1950) *Rec. Progr. Horm. Res.*, **5**, 383.—(1953) *Anat. Rec.*, **115**, 381.—(1954) *J. nat. Cancer Inst.*, **15**, 717.
*Idem* AND LITTLE, C. C.—(1946) *Cancer Res.*, **6**, 712.